# The effects of tea plants-soybean intercropping on the secondary metabolites of tea plants by metabolomics analysis

**DOI:** 10.1186/s12870-021-03258-1

**Published:** 2021-10-22

**Authors:** Yu Duan, Xiaowen Shang, Guodong Liu, Zhongwei Zou, Xujun Zhu, Yuanchun Ma, Fang Li, Wanping Fang

**Affiliations:** 1 College of Horticulture, Nanjing Agricultural University, Nanjing, 210095 China; 2 Department of Plants Science, University of Manitoba, 66 Dafoe Road, Winnipeg, MB R3T 2N2 Canada

**Keywords:** Tea plants-soybean intercropped, Secondary metabolites, Amino acids metabolites, Metabolic pathway

## Abstract

**Background:**

Intercropping, especially with legumes, as a productive and sustainable system, can promote plants growth and improves the soil quality than the sole crop, is an essential cultivation pattern in modern agricultural systems. However, the metabolic changes of secondary metabolites and the growth in tea plants during the processing of intercropping with soybean have not been fully analyzed.

**Results:**

The secondary metabolomic of the tea plants were significant influence with intercropping soybean during the different growth stages. Especially in the profuse flowering stage of intercropping soybean, the biosynthesis of amino acids was significantly impacted, and the flavonoid biosynthesis, the flavone and flavonol biosynthesis also were changed. And the expression of metabolites associated with amino acids metabolism, particularly glutamate, glutamine, lysine and arginine were up-regulated, while the expression of the sucrose and D-Glucose-6P were down-regulated. Furthermore, the chlorophyll photosynthetic parameters and the photosynthetic activity of tea plants were higher in the tea plants-soybean intercropping system.

**Conclusions:**

These results strengthen our understanding of the metabolic mechanisms in tea plant’s secondary metabolites under the tea plants-soybean intercropping system and demonstrate that the intercropping system of leguminous crops is greatly potential to improve tea quality. These may provide the basis for reducing the application of nitrogen fertilizer and improve the ecosystem in tea plantations.

**Supplementary Information:**

The online version contains supplementary material available at 10.1186/s12870-021-03258-1.

## Background

Tea plants (*Camellia sinensis* (L.) O. Kuntze) are cultivated worldwide as an economical woody plant [1]. The young fresh leaves of tea plants contain abundant secondary metabolites, for example, polyphenols, amino acids, caffeine, organic acids as well as vitamin, and so on [2, 3]. These secondary metabolites can affect the tea quality, and be influenced by agronomy management in the meantime [4–6].

One-third of all the cultivated land area is used for multiple cropping and half of the total grain yield is produced with multiple cropping in China [7]. Intercropping, as one of the multiple cropping systems, is important to subsistence agriculture or low-input/ resource-limited agricultural system [8, 9]. Intercropping is focused on the plants-plants interactions for light, optimal temperatures and space above-ground [10]. Recently, some studies had reported the below-ground interaction including complementary interactions between crop plants and soil microbe to explore the impact on the resource use efficiency and crops yield and quality [8, 11–15]. There are many intercropping patterns, such as tree/crop and crop/crop [16–19], that increased crop yields and soil nutrient availability [20].

Most studies were focused on the legume-cereal intercropping, and the intercropping systems were restricted nitrogen supply, and legumes could increased N availability and agricultural productivity [21]. The legumes are less competitive than other plants in absorbing nitrogen from the soil, and can nitrogen-fixing by their root nodules and contribute up to 15% of the N in an intercropped cereal [22, 23]. Besides, the non-legumes obtain additional nitrogen from that released by legumes into the soil [15, 24]. We have noticed that the cereal–legume intercropping systems, the shorter and more shaded legume will capture solar radiation more efficiently in the intercropped than monoculture [25].

Previous studies have shown that chestnut-tea plants intercropping in the tea plantations can affect the microclimate, reduce soil bulk density and soil erosion [26]. That also be improved soil nutrients and moisture such as increases soil carbon and nutrient availability [27, 28]. Besides, the intercropping also can increase tea buds length, the weight of one hundred buds and the content of theanine, and then improves the tea quality and yield [29, 30]. These studies were mainly focused on the impacts of the intercropping modern on soil nutrients and enzyme activities in tea plantation. However, in the intercropping system of tea plants-legumes, the identification and quantification of metabolites with the tea quality, elucidation of metabolic pathways, and the regulation of these pathways remains unclear.

Non-targeted metabolomics involves the simultaneously unbiased detection of a huge range of endogenous metabolites, analyzes the pathways of the differential metabolites, and reveals the physiological mechanism of living organisms. The non-targeted metabolomics was applied in tea plants to detect more compounds using GC-MS or LC-MS [31–33]. In our previous study, in the tea plants-soybean intercropping system, the content of the amino acids in intercropped tea plants was significantly increased, and the contents of other secondary metabolites also were changed, besides, the content of nitrogen in the intercropped soil was increased [34]. Thus, the non-targeted metabolomics approach was used to analyze metabolic profile changes of the tea leaves during the different growth stages of soybean in the tea plants-soybean intercropping system. Our study indicates that when intercropping soybean growth to the profuse flowering stage, the flavonoid, amino acids, the carbohydrate as well as the compounds associated with their metabolism in tea leaves were significantly changed, which could promote the tea plants growth and improve the tea quality finally.

## Methods

### Plants materials and growth conditions

The young tea plants with the annual ‘Su Cha Zao’ tea cultivar growing in the tea company of Nanjing Yarun(Nanjing Yarun Tea Industry Co. Ltd., Nanjing, China) and the soybean with high insect resistance and drought resistance of variety ‘Lamar’ came from National Soybean Improvement Center of Nanjing Agricultural University were acquired from Professor Fajun Chen lab, College of Plant Protection, Nanjing Agricultural University. The young tea plants were intercropped with soybean in the greenhouse. The monoculture treatment was designed as just young tea plants, and the rows were spaced to 40 cm and the distance between tea plants in a row was 15 cm. When the young tea plants growth to steady state and healthy, the soybean seeds were sown in-row spacing (20 cm) between tea plants which was the intercropped treatment. A bud as well as first and second leaves from the top bud from the each tea plants were carefully collected when the intercropped soybean growth to seedling, profuse flowering, and mature stages. There were three biological replicates were performed for each sample. All collected samples were immediately stored at − 80 °C liquid nitrogen to minimize chemical changes during storage before analysis.

### Chemicals and reagents

All chemicals and reagents were analytical grades. Methyl alcohol, acetonitrile, and ethyl alcohol were purchased from Merck Company, Germany (www.merckchemicals.com). Milli-Q system (Millipore Corp., Bedford, MA, USA) ultrapure water was used throughout the study. Authentic standards were purchased from BioBioPha Co., Ltd. (www.biobiopha.com/) and Sigma-Aldrich, USA (www.sigmaaldrich.com/unitedstates.html).

### The photosynthetic and chlorophyll fluorescence parameters were determined in tea plants leaves

The parameters of photosynthetic and chlorophyll fluorescence were determined by Li-6400 automatic portable photosynthesizer (LI-COR, USA) and PocketPEA Portable fluorometer (Hansatech, UK), respectively. The second leaves from the top bud were used for the measurements. When the soybean growed to the seedling stage, at 9:30, 10:30, 11:30, 12:30, 13:30, 14:30, 15:30 and 16:30 on the day, the real-time illumination were 700 μmol· m^− 2^ ·s^− 1^, 900 μmol· m^− 2^ ·s^− 1^, 1000 μmol· m^− 2^ ·s^− 1^, 1500 μmol· m^− 2^ ·s^− 1^, 1300 μmol· m^− 2^ ·s^− 1^, 1200 μmol· m^− 2^ ·s^− 1^, 700 μmol· m^− 2^ ·s^− 1^ and 500 μmol· m^− 2^ ·s^− 1^, respectively. And the photosynthetic parameters of tea plants under the soybean different stages were measured with the above illuminance. All tests were performed 15 biological repeats and 3 technical repeats.

### Extraction and fractionation

The frozen tea samples were sonicated in liquid nitrogen with a zirconia bead for 1.5 min at 30 Hz using a mixer mill (MM 400, Retsch). Then a total of 100 mg powder was weighed and extracted with 1 mL of methanol/water (7:3, v/v) containing 0.1 mg/L lidocaine for the internal standard and incubated overnight at 4 °C. Following centrifugation at 10,000 g for 10 min, the supernatant was absorbed and filtrated (SCAA-104, 0.22-μm pore size; ANPEL, Shanghai, China, www.anpel.com.cn/) into autosampler vials for LC-MS analysis [35].

### Data analysis

Data analyses were performed with GraphPad Prism 7.0 (GraphPad Software, Inc., San Diego, CA) and SigmaStat Software (SPSS, Chicago, IL, USA). All data were expressed as the mean values±S.D. Significant differences were determined by One-way ANOVA.

Metabolites were identified by searching the internal database and public databases (MassBank, KNApSAcK, HMDB, MoTo DB, and METLIN) and comparing the m/z values, RT, and the fragmentation patterns with the standards [36]. As the derivative algorithm of PLS-DA, the orthogonal partial least-squares discriminant analysis (OPLS-DA) was used to visualize the dissimilarity/distinction among experimental samples. Following the multivariate analysis, the significance of each metabolite in-group discrimination was further evaluated using *t*-tests values, and the *p* < 0.05 and VIP ≥ 0.8 were considered to indicate the significant difference variable in the PLS-DA analysis. Differential metabolites between the different treatments of tea samples in the different growth stages of soybean were analyzed using the KEGG pathway database in channel enrichment [37].

## Results

### The parameters of photosynthetic and chlorophyll fluorescence in the tea plants of monoculture and intercropping during the different growth stages of soybean

**Fig. 1:**
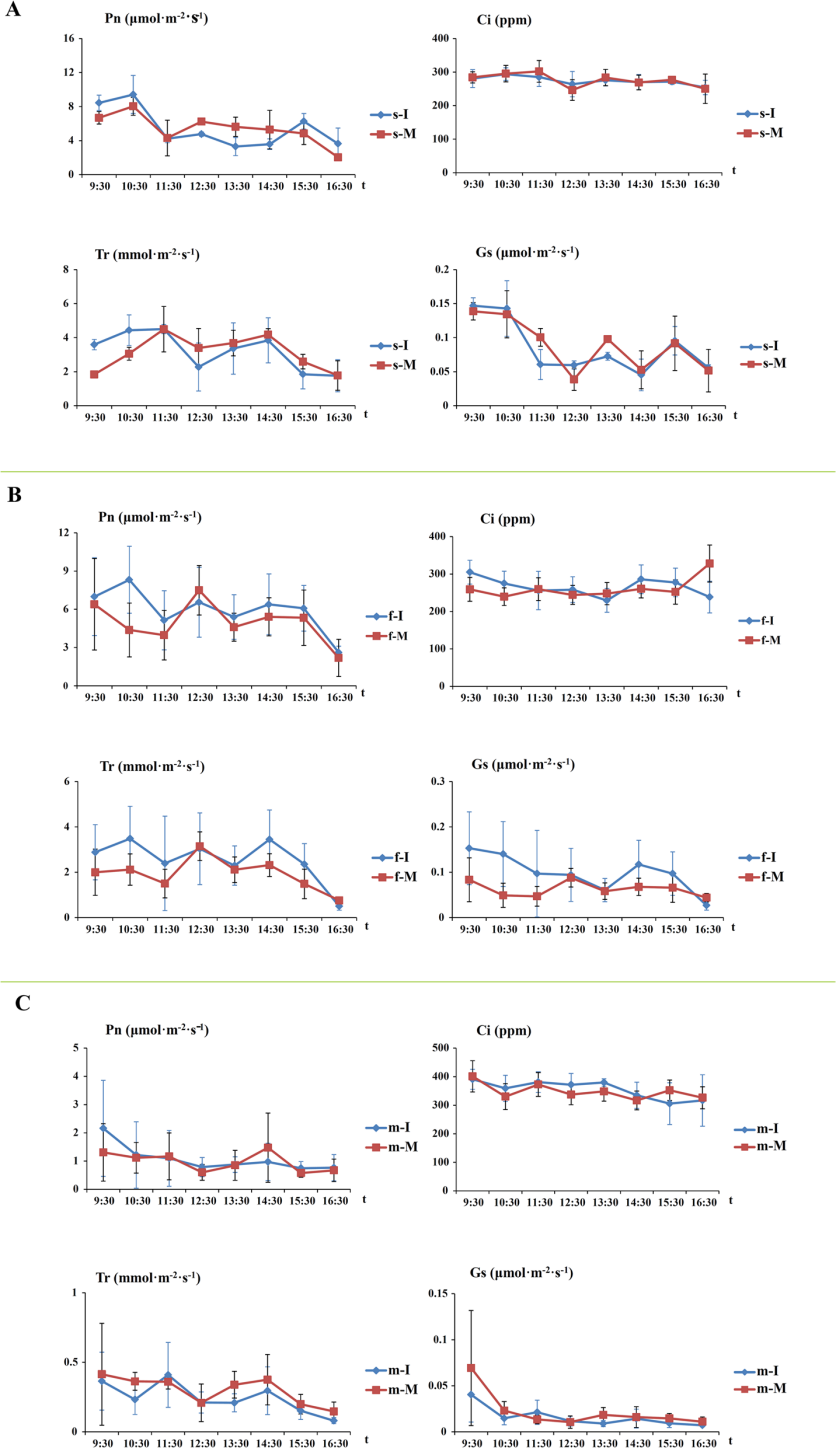
Effects of intercropping on the parameters of photosynthetic in *C.sinensis* leaves. (**A**) The photosynthetic parameters in tea plants during the soybean seedling stage (**B**) The photosynthetic parameters in tea plants during the soybean profuse flowering stage (**C**) The photosynthetic parameters in tea plants during the soybean mature stage. I indicates intercropping; M indicates monoculture; s indicates seedling stage of soybean; f indicates profuse flowering stage of soybean; m indicates mature stage of soybean

Using the Li-6400 automatic portable photosynthesizer analyzed the photosynthetic parameters revealed that the tea plants-soybean intercropped were beneficial to the growth of tea plants (Fig. 1A, B, C). There was no significant difference in stomatal conductance (Gs) of intercropped and monoculture young tea plants in the soybean seedling and maturity stages. However, during the soybean profuse flowering stage, the stomatal conductance (Gs) of the intercropping tea plant was significantly higher than the monoculture tea plants. We also found that the stomatal conductance of intercropped tea plant leaves was slightly larger during 12:30–13:30 at noon (Fig. 1B). The net photosynthetic rate (Pn) and transpiration rate (Tr) of the intercropping tea plants were higher during the soybean profuse flowering stage at 12:30 noon. However, during the soybean seedling stage and mature stage, intercropping tea plants’s net photosynthetic (Pn) and transpiration rate (Tr) were relatively lower after 11:30. Besides, accompanying soybean growth and development, the Gs, Pn and Tr of tea plants were significantly reduced in both the monoculture and intercropping tea plants during the soybean mature stage.

**Fig. 2:**
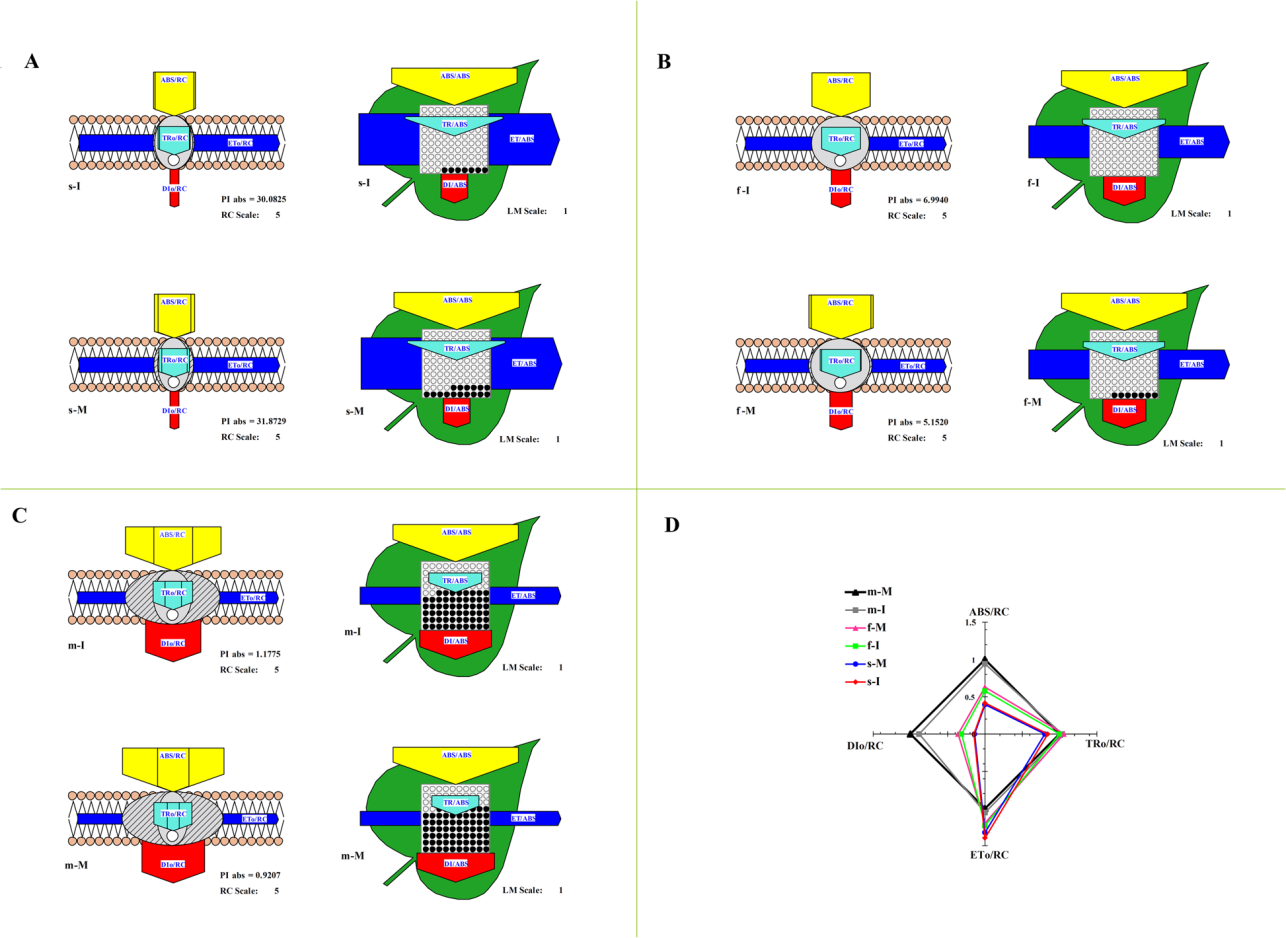
Effects of intercropping on the chlorophyll fluorescence parameters in *C.sinensis* leaves. (**A**) The pipeline models for phenomenological fluxes (leaf model) or specific fluxes (membrane model) in tea plants during the soybean seedling stage (**B**) The pipeline models for phenomenological fluxes (leaf model) or specific fluxes (membrane model) in tea plants during the soybean flowering-podding stage (**C**) The pipeline models for phenomenological fluxes (leaf model) or specific fluxes (membrane model) in tea plants during the soybean mature stage (**D**) Parameters of fast chlorophyll fluorescence induction kinetics curve in tea plant during the soybean growth stage. I indicate intercropping; M indicates monoculture; s indicates seedling stage of soybean; f indicates profuse flowering stage of soybean; m indicates mature stage of soybean

To better understand the differences in energy flow in photosynthesis between monoculture and intercropping systems, the specific membrane models were compared in tea plants (Fig. 2A, B, C, and D). In soybean seedling and the mature stages (Fig. 2A, C), the tea plants of intercropping and monoculture has no significant difference in light energy absorption yield (ABS), electron transfer rate (ET) and heat dissipation (DI), and in the soybean mature stage, there was a slight difference in the opening ratio of the PSII reaction center. While in the soybean profuse flowering stage (Fig. 2B), the light energy absorption (ABS) and heat dissipation (DI) were higher in the intercropped tea plants, and the maximum photochemical efficiency (TR/ABS) showed no significant difference, and the charge separation ability of the reaction center was stronger with intercropping tea plants. In addition, to explore the energy flow condition in photosynthesis, the tea plants were as the reference (normalized to 1) at the soybean mature stage and performed the radar graphs of different JIP-test parameters, including active unit response center (per RC) and light area per unit (per CS) (Fig. 2D). We found that the light energy absorbed per unit reaction center (ABS/RC) and energy dissipated per unit reaction center (DI/RC) of the plants in soybean mature stage were much higher than that of soybean seedling and profuse flowering stage (Fig. 2D). However, the light energy absorbed per unit area (ABS/CSo) and heat dissipation per unit area (DI/CSo) of tea plants in the soybean profuse flowering stage were the highest and lowest, respectively, indicating that tea plants absorbed light energy with the highest rate and the strongest in charge separation ability of photosynthesis. The PSII reaction center opening ratio (RC/CSo), electron transport efficiency (ETo/RC, ETo/CSo) in the tea plants of intercropping were higher than monoculture. Meanwhile, the heat dissipation (DIo/RC and DIo/CSo) was significantly lower (Fig. 2D).

### Multivariate statistical analysis of metabolites in tea plants of monoculture and intercropped during different growth stages of soybean

**Fig. 3:**
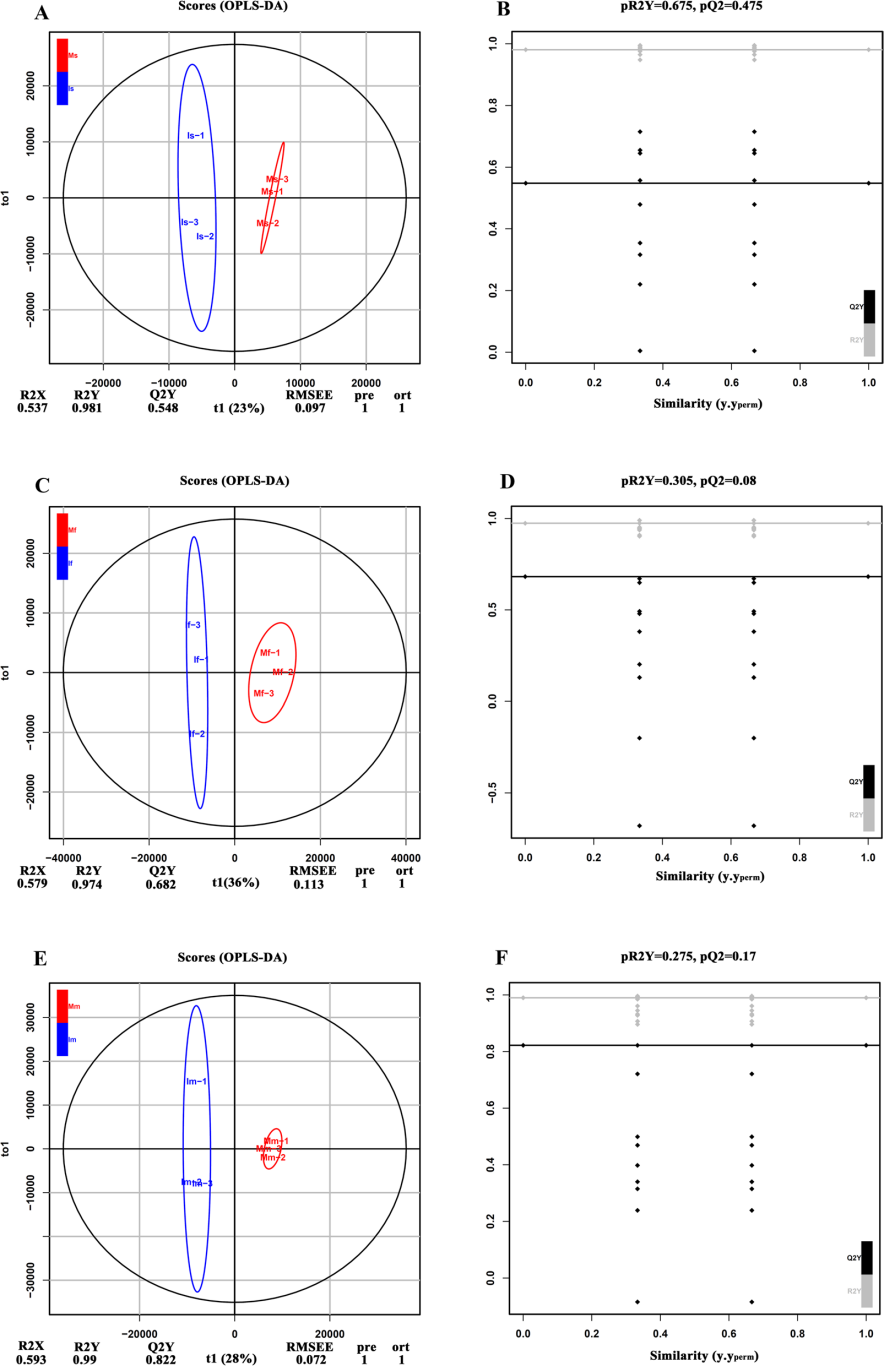
OPLS-DA score plots (**A**, **C** and **E**) and permutation test (**B**, **D** and **F**) derived from GC-MS data from the tea plants. A and B indicates the soybean seedling stage; C and D indicates the soybean profuse flowering stage; **E** and **F** indicates the soybean mature stage; I indicates intercropping; M indicates monoculture

To probe into the significantly correlated metabolites on tea plants of monoculture and intercropped, the orthogonal partial least-squares discriminant analysis (OPLS-DA) modeling was applied to LC-MS data sets, and the accuracy of OPLS-DA models was evaluated by the sequencing test (Fig. 3). In the soybean seedling stage, the monoculture treatment was separated from the intercropped by the predictive component t (1) (23%), and the parameters for the OPLS-DA model validated, such as the fitness (R2X = 0.537 and R2Y = 0.981), predictability (Q2 = 0.548), and permutation values (R2 and Q2 intercepts = 0.675 and 0.475, respectively) (Fig. 2A and B). In the soybean profuse flowering stage, the treatment group of Mf-vs-If was distinguished by the predictive component t (1) (36%). We observed the parameters for the OPLS-DA model, and included the fitness (R2X = 0.579 and R2Y = 0.974), predictability (Q2 = 0.682), and permutation values (R2 and Q2 intercepts = 0.305 and 0.08, respectively) (Fig. 3C and D). In the soybean mature stage, the treatment group of Ms-vs-Is was identified by the predictive component t (1) (28%), and we observed the parameters for the OPLS-DA model, included that the fitness (R2X = 0.593 and R2Y = 0.99), predictability (Q2 = 0.822), and permutation values (R2 and Q2 intercepts = 0.275 and 0.17, respectively) (Fig. 3E and F). These results indicated that the models were stable and reliable as a predictable model.

### Metabolic changes in tea plants of monoculture and intercropped during different growth stages of soybean

**Fig. 4:**
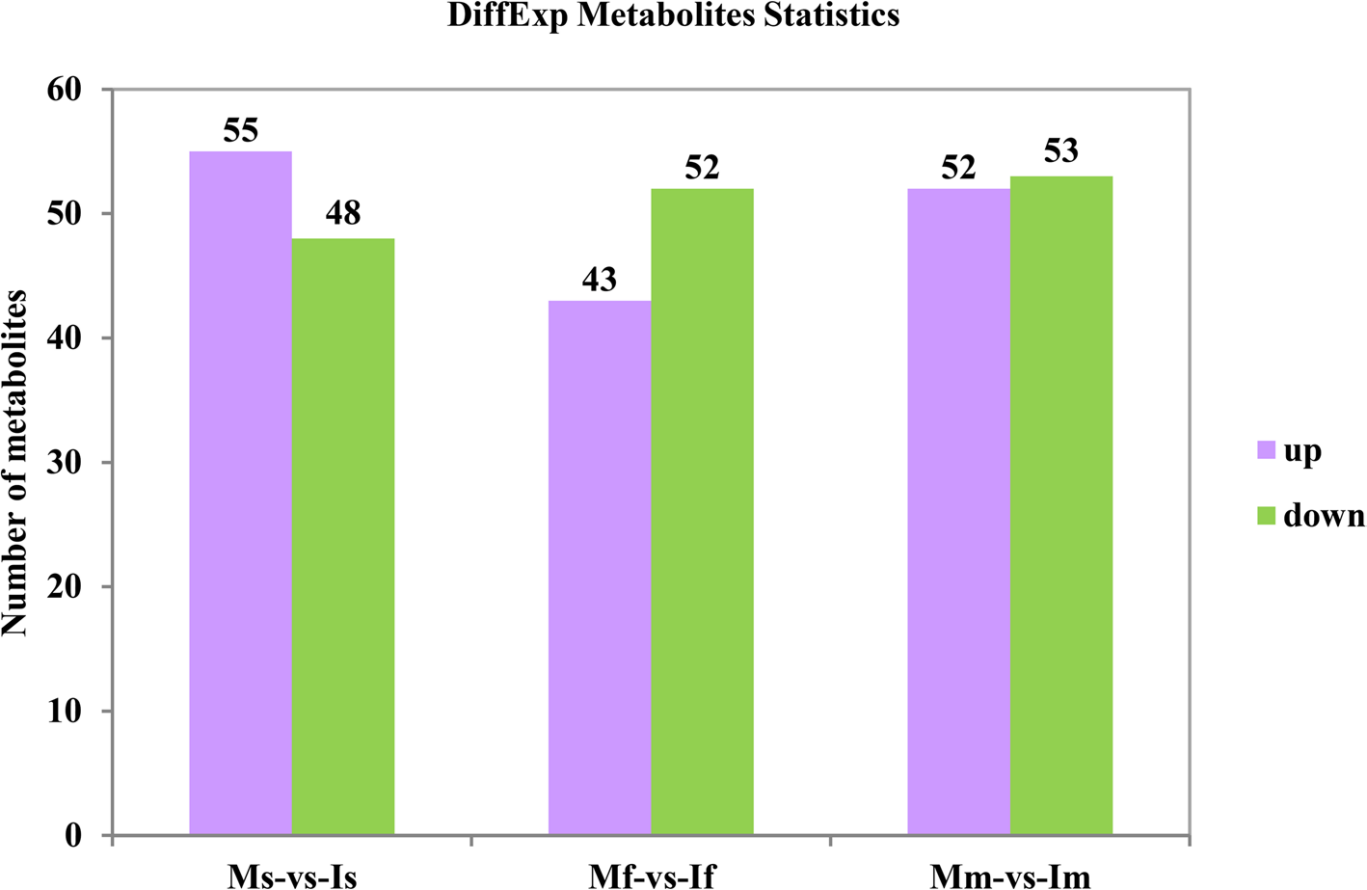
The significantly differential metabolites in the tea plants of monoculture and intercropped. The threshold of significant difference was VIP ≥ 0.8 and T-test *P* < 0.05. I indicate intercropping; M indicates monoculture; s indicates seedling stage of soybean; f indicates profuse flowering stage of soybean; m indicates mature stage of soybean

**Fig. 5:**
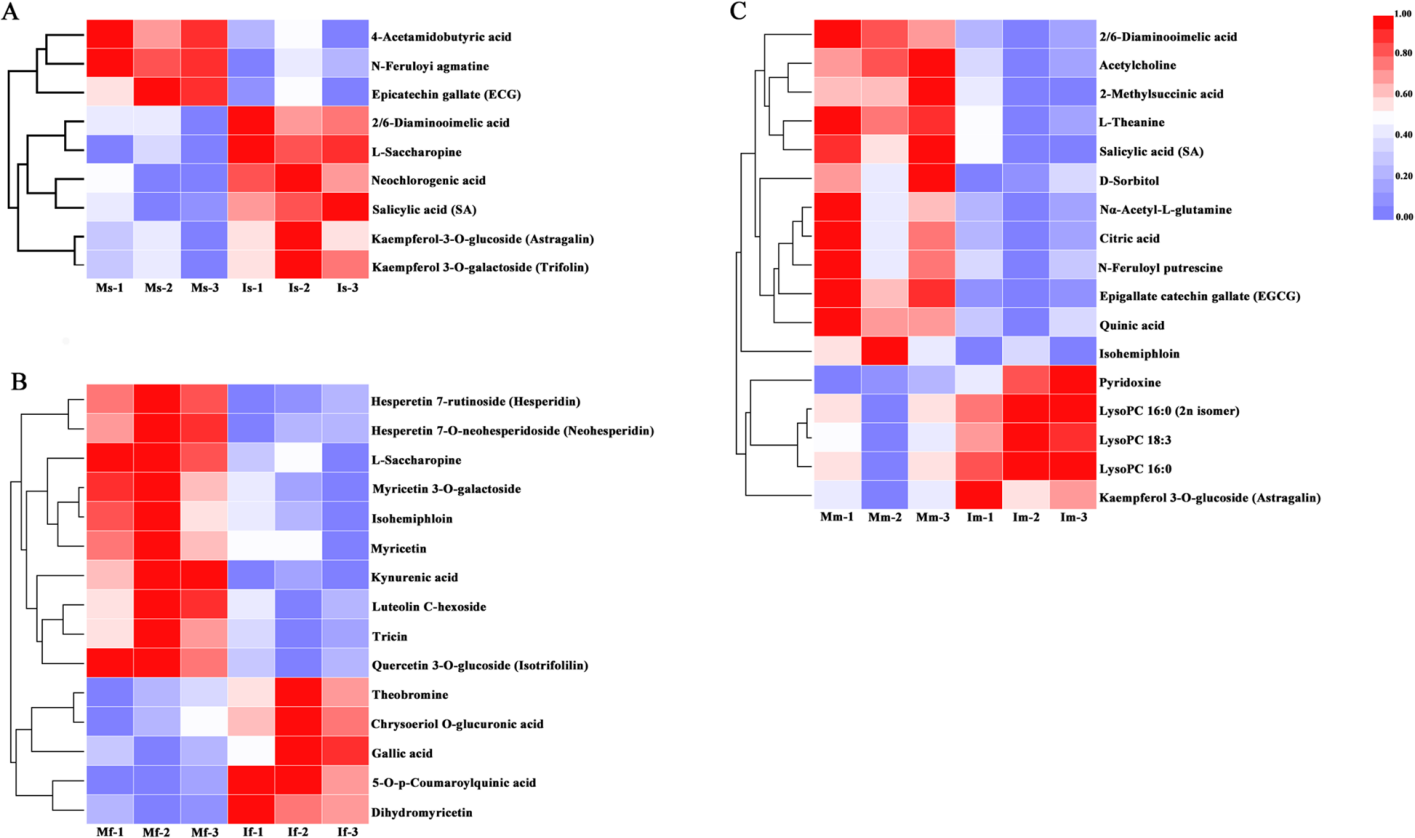
Heat map of the identified differential metabolites from the tea plants of monoculture and intercropped. I indicates intercropping; M indicates monoculture; s indicates seedling stage of soybean; f indicates profuse flowering stage of soybean; m indicates mature stage of soybean

In different growth stages of soybean, compared the tea plants of monoculture, we found that the total 103 differential metabolites were annotated in Ms-vs-Is, and 55 and 48 were up-regulated and down-regulated, respectively. A total of 95 differential metabolites were annotated in Mf-vs-If, including that 43 and 52 were up-regulated and down-regulated, respectively. In addition, 105 differential metabolites were annotated in Mf-vs-If, of which 52 were up-regulated and 53 were down-regulated, respectively (Fig.4). Besides, the metabolites of the all treatments in soybean different growth stages were standardized [0, 1] by min-max, and carried out the cluster analysis and heat map. The metabolites of all treatments mainly contained amino acids and their derivatives, flavone, flavonol, phytohormones. The different metabolites in soybean seedling were much less than the other two growth stages of soybean (Fig.5A). Furthermore, in the soybean profuse flowering stage, the metabolites were mainly classified as flavone, flavonol and flavanone (Fig.5B). The expression of these metabolites was relatively lower in intercropping. While in the soybean mature stage, the metabolites were categorized to many compounds, and most of their expression was relatively lower in intercropped tea plants (Fig.5C and Supplementary Table. S1).

### Enrichment analysis of different metabolites in KEGG metabolic pathways for tea plants of monoculture and intercropped during different growth stages of soybean

**Fig. 6:**
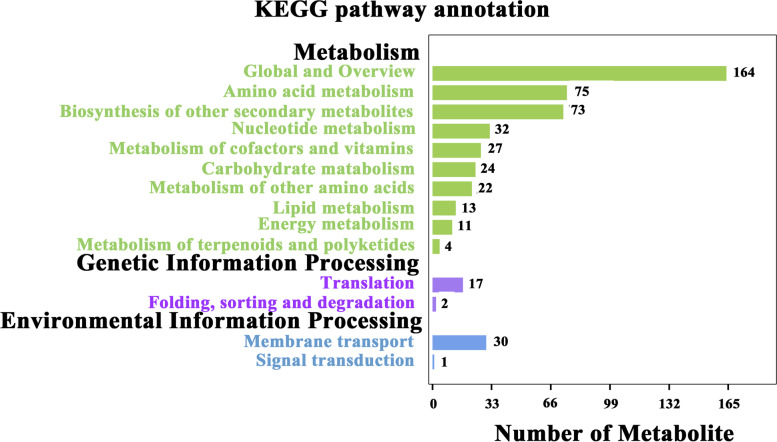
Distribution chart showing the differential metabolites of various metabolic pathways for the tea plants

**Fig. 7:**
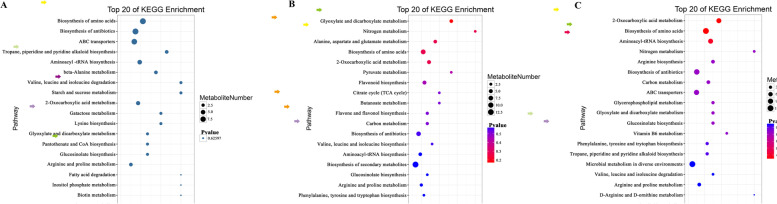
The bubble map of metabolite pathway enrichment analysis in the tea plants of monoculture and intercropped. **A** indicates the Ms-vs-Is (soybean seedling stage); **B** indicates the Mf-vs-If (soybean profuse flowering stage); **C** indicates the Mm-vs-Im (soybean mature stage)

By the enrichment and analysis of the KEGG pathway, the results were showed that the differential metabolites were involved in various metabolic pathways (Fig.6). The classes of metabolism (MB) were mainly contained the metabolic pathway of amino acids metabolism (75), biosynthesis of other secondary metabolites (73), nucleotide metabolism (32), carbohydrate metabolism(24) and the metabolism of other amino acids (22). Both the genetic information processing (GIP) and environmental information processing (EIP) contained two metabolic pathways, which the amino acids accounted for 43.3% of the metabolites involved in membrane transport of the EIP (Table S2). During the soybean seedling stage, the max value of Rich Factor was 0.5 and the *p*-value in the top 20 metabolism pathway of the KEGG enrichment were all 0.62597. These results showed that the enrichment of the metabolism for tea plants in monoculture and intercropping was low, and no significant difference (Fig. 7A). However, during the soybean profuse flowering and mature stages, the KEGG enrichment for the most of top 20 metabolism pathways was significantly changed, such as and the nitrogen metabolism, biosynthesis of amino acids, the 2-Oxocarboxylie acid metabolism and carbon metabolism, and so on (Fig.7B and C). In addition, at the soybean profuse flowering stage, we found the nitrogen metabolism and biosynthesis of amino acids were predominant, and some metabolism pathways only were found, including that the alanine, aspartate and glutamate metabolism, the valine, leucine and isoleucine biosynthesis, the biosynthesis of secondary metabolites, flavonoid biosynthesis as well as flavone and flavonol biosynthesis. While the valine, leucine and isoleucine degradation were not found (Fig.7B).

### Correlation analysis of metabolites in tea plants of monoculture and intercropped during different growth stages of soybean

**Fig. 8:**
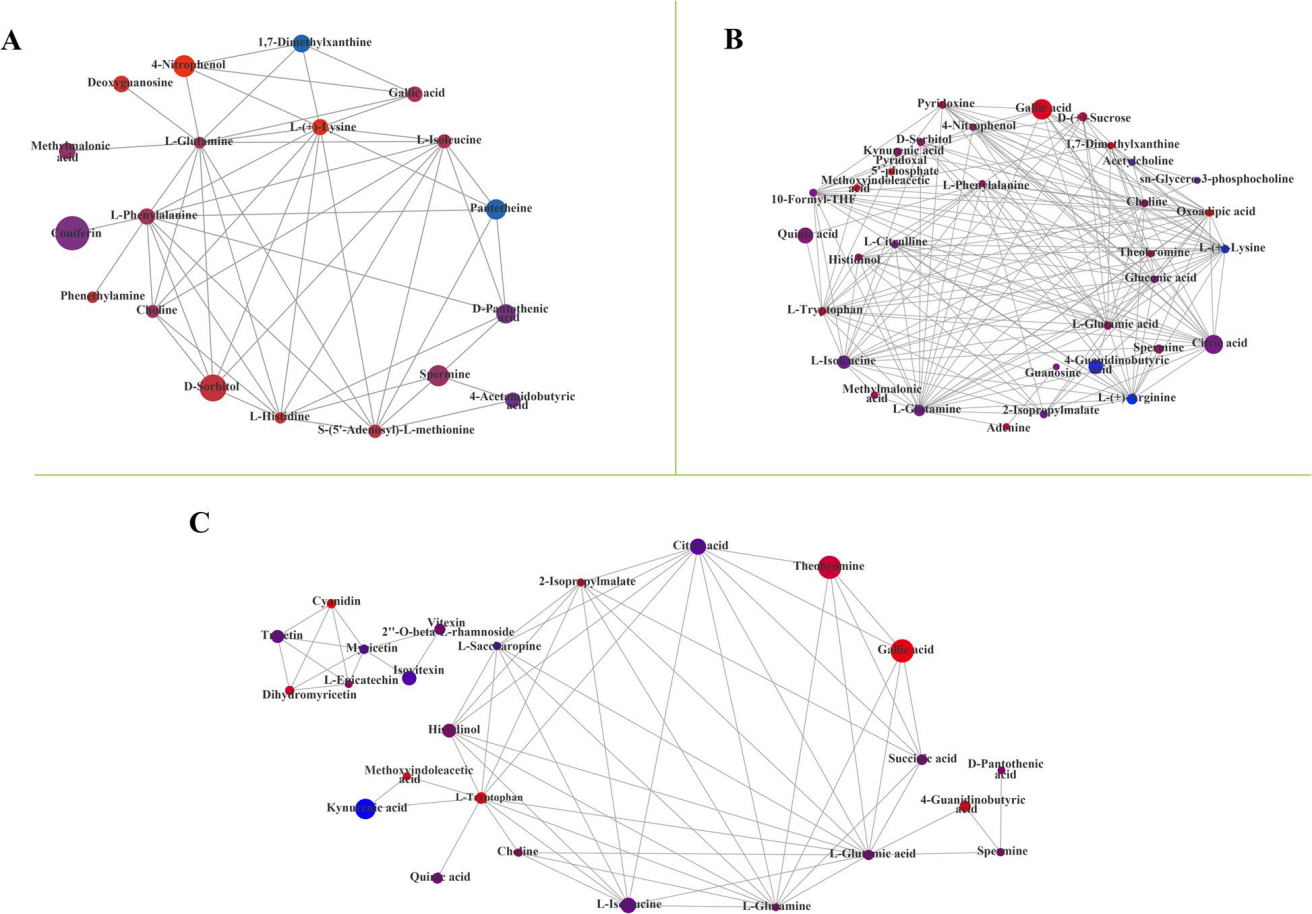
Pathway enrichment analysis of the primary pathway-associated differential metabolite sets. In these networks, the node indicates the correlated metabolisms, and the lines between two nodes represent the biological relationships between two metabolites. The size of the node indicates the VIP value, and the color indicates the log_2_FC. The lines represented the metabolic pathway. A indicates the soybean seedling stage; B indicates the soybean mature stage; C indicates the soybean profuse flowering stage

The association network diagram of metabolites and metabolic pathways was plotted by Cytoscape analysis software, and the differential metabolites in tea plants of monoculture and intercropped during soybean different growth stages were integrated. As depicted in Fig.8, the result was revealed that the metabolites interacted and coordinated the regulation of multiple metabolic pathways. Almost all metabolites were presented in the same metabolic pathway in these metabolites in the soybean mature stage (Fig.8B). Whereas, in the seedling and profuse flowering stages of soybean, every two or more metabolites exist in the same metabolic pathway and also the metabolite participates in multiple metabolic pathways (Fig.8A and C). Furthermore, the differential metabolites in tea plants of monoculture and intercropped formed two complete network metabolites in the profuse flowering stage of soybean, and the flavonol, flavone C-glycosides and the catechin derivatives were constituted the completed metabolic network diagram, and only one complete metabolite network was formed in the other two growth stages of soybean (Fig. 8).

### Metabolic characteristic of amino acids and sucrose metabolites in the tea plants of monoculture and intercropped during different growth stages of soybean

**Fig. 9:**
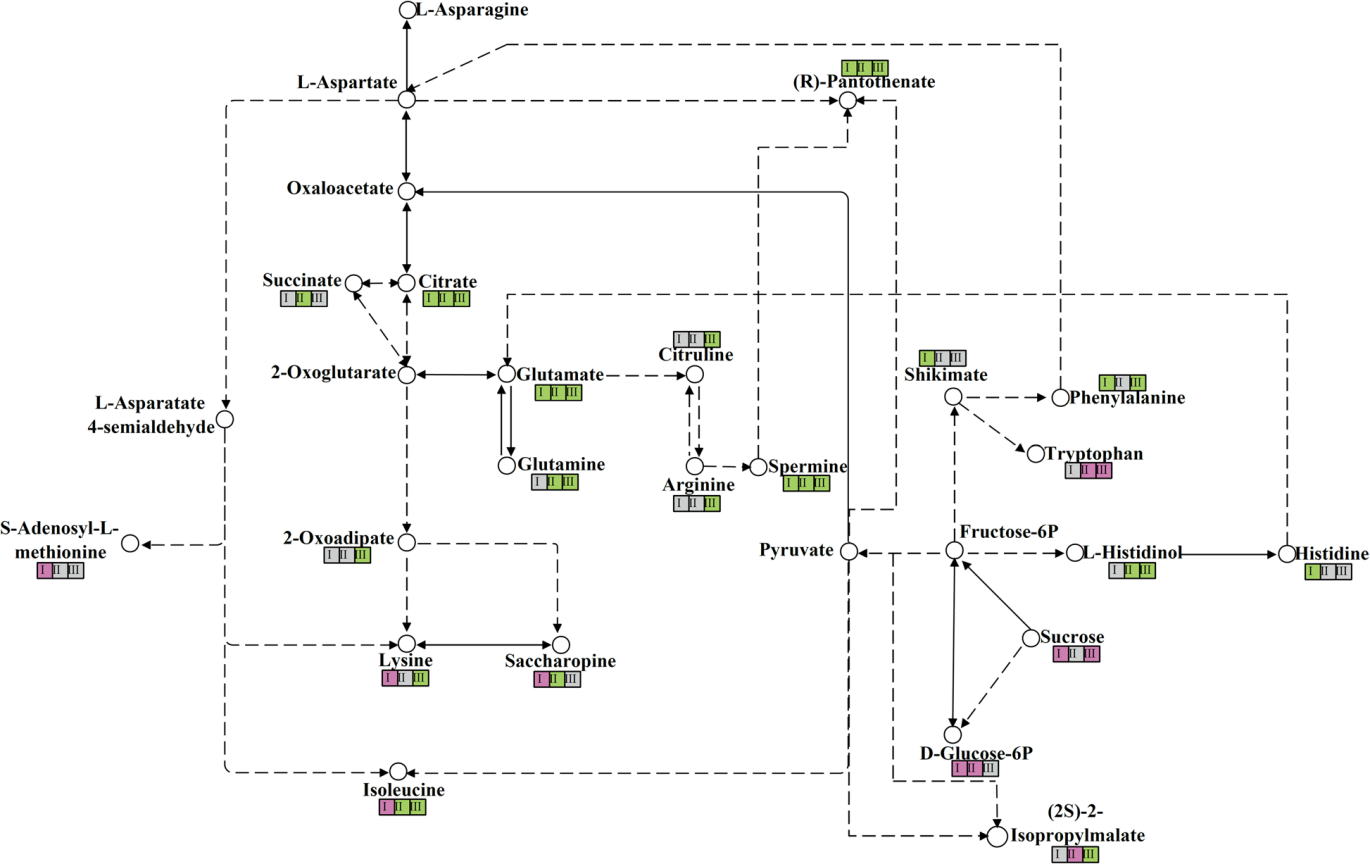
Metabolism of amino acids and sucrose in the tea plants of monoculture and intercropped. Red represents up-regulation; green represents down-regulation; I indicates the M-vs-I during the seedling stage of soybean; II indicates the M-vs-I during the profuse flowering stage of soybean; III indicates the M-vs-I during the mature stage of soybean

In this study, compared to the monoculture tea plants, the different metabolites were observed during the different growth stages of soybean. The metabolites related to amino acids and carbohydrates biosynthesis were found and selected during the three growth stages of intercropping soybean, and according to the KEGG pathway with www.kegg.jp/kegg/kegg1.html, their relationships were described in Fig.9. According to the metabolic diagram, all metabolites of tea plants related to amino acids were down-regulated during the stage of soybean profuse flowering and mature with M-vs-I. However, these metabolites were up-regulated or down-regulated in the soybean seedling stage. Also, the number of metabolites related to carbohydrates was relatively fewer and these metabolites were up-regulated in soybean different growth stages.

## Discussion

Photosynthesis was necessary for the tea plants’ growth and metabolism and obviously promoted the production and improved the tea quality. As we know, the diurnal variation of photosynthetic parameters could reflect the daily photosynthetic production in plants [38, 39]. In the soybean profuse flowering stage, the tea plants photosynthetic parameters including Pn, Tr and Gs, were significantly increased. Due to the soybean with the profuse flowering stage was vigorous growth, and formed the moderate shading effect on young tea plants. Therefore, intercropped soybean in tea plantations could promoted tea plants’ growth and development, especially in the soybean profuse flowering stage. And moderate shading of the soybeans could impact and promote the growth of young tea plants [30].

The changes of chlorophyll fluorescence were closely associated with photosynthetic reactions, and the chlorophyll fluorescence parameters could effectively reflected photosynthetic performance characteristics [40]. The pipeline models for phenomenological fluxes (leaf model) or special fluxes (membrane model) of the tea plant, as well as the chlorophyll photosynthetic parameters, indicating that the photosynthetic activity of tea plants in the soybean profuse flowering stage was higher, and the PI_ABS_ of tea plants with intercropping soybeans were increased. Because the chlorophyll fluorescence parameters were shown the plants` photosynthetic apparatus and capacity, and the light was very important source for the regulation of plants growth and development [30, 41]. That is, in tea plantations, the intercropping soybeans with the profuse flowering stage were more beneficial to the growth and development of tea plants.

In the previous studies, the metabolomic techniques were used to detect various metabolites simultaneously or compare samples reliably to identify differences and similarities [30, 42]. And the effects of intercropped legumes with tea plants were analyzed the metabolisms of tea plants in this study. In the present study, the secondary metabolites plants at soybean different growth stages were different between intercropped and the monoculture tea plants. Under the profuse flowering stage, the different metabolites were more abundant, and the flavonoid compounds were relatively low in intercropping tea plants. While most of the secondary metabolites including the amino acids, alkaloids, organic acids, phytohormones as well as polyphenols, and so on, were less abundant at the mature stage. Due to the secondary metabolites of tea plants, including flavonoids, polyphenols, theanine, alkaloids, and aroma compounds were identified [43, 44]. These secondary metabolites were closely related to tea quality and nutritional and healthy function [45, 46]. And the intercropped legumes affected the nitrogen input from the soil, and increase agricultural productivity, the intercropping nonleguminous plants could obtain additional nitrogen by the legume [21, 47]. The soil nitrogen could be transported or uptake by plants, and produced different forms of the nutrient compounds [16, 48]. The low polyphenol content and high amino acids level of tea plants could significantly reduce the taste of bitterness and astringence, increase the umami flavor of tea infusion, and furtherly improve the tea quality [49]. Therefore, our study suggested that the soybean intercropped could affect the growth condition of tea plants and the kinds of secondary metabolites, further improved the tea quality, especially in the soybean profuse flowering stage [48]. In the top 20 of KEGG enrichment, the metabolisms of amino acids and nitrogen were significantly enriched. The amino acids are potential N sources for plants and are influenced by plants’ variety growth environments. Furthermore, the biosynthesis of amino acids requires nitrogen from the soil, and nitrogen nutrition is essential for amino acids synthesis in plants [50, 51]. Many studies have reported the amino acids are closely related to the nutrient uptake in the plants’ roots. Because the intercropping of leguminous plants could increased the plants’ nutrient supply by stimulation of biological nitrogen fixation in legume rhizobia symbiosis to increase the nitrogen availability [52]. At the same time, the perennial grains intercropped with the legume could be an option, as the legume could enhance facilitative roots and microbial processes to create a more stable agroecosystem [53]. In the present study, we also found that the amino acids metabolism pathways with map 00250, map 00290, map 002330, map 00240, and the amino acids metabolites were obviously different with the tea plants-soybean intercropped system, especially when the soybean developed to the profuse flowering stage. In addition, the intercropped soybean for the profuse flowering stage also had an influence in the flavonoid biosynthesis and the flavone and flavonol biosynthesis. This may because the nitrogen fixation of the leguminous crops was affected the phenolic compounds, and further facilitated the absorption of nutrients [54]. Therefore, the intercropping of soybean could influenced or promoted the synthesis of tea plants secondary metabolites, especially the amino acids during the soybean profuse flowering stage.

The tea is made from the tender leaves of the tea plants (*Camellia sinensis* (L.) O. Kuntze), and is one of the three most popular consumed nonalcoholic beverages. Previous studies focused on the relationships of tea metabolites with tea quality and function [55–57]. In this study, during the profuse flowering stage of intercropping soybean, the different metabolites were involved in the different metabolic pathways and and formed two complete metabolism network diagrams as the flavonoid compound metabolism and other all secondary metabolites metabolism. Furthermore, we also found the expression of metabolites associated with amino acids metabolisms, such as the glutamate, glutamine, lysine and arginine were up-regulation, while the expression of the sucrose and D-Glucose-6P related to carbohydrate metabolism were down-regulated in the intercropping soybean profuse flowering and mature stages. The reasons might be the intercropping of soybean could increased the soil inorganic nitrogen, and then was assimilated into amino acids in plants roots and/or leaves, then the amino acids in plants roots also were transported to the plant leaves [58]. And the the inorganic nitrogen by soybean nitrogen fixation was absorbed to tea plants and were assimilated into amino acids [59]. In addition, the amino acids, flavonoids, and carbohydrates, as well as their metabolism were essential to the tea plants growth and development, and affected the tea quality formation, especially the glutamate, glutamine and arginine [60, 61]. It should be noted that intercropping soybean with tea plants was beneficial to the tea plants’ growth, and improved the tea quality, especially when soybeans grow to the profuse flowering stage. And these study results were consistent with the previous basic research [34].

## Conclusion

The metabolites of tea plants were analyzed in intercropped soybean’s different growth stages by metabolomics and combined with the photosynthesis fluorescent parameters to further summarized the effects of intercropped soybean on the tea plants’ growth and development. The results showed the intercropping with soybean was affected the synthesis and metabolism of amino acids, further improving the tea quality, especially when the intercropping soybean growth to the profuse flowering stage. Thus, the intercropping system potential influences on tea plantation cultivation management were worthy of further exploring. It might provide the basis for reducing the application of nitrogen fertilizer in tea plantations, and also be beneficial to improve the sustainability and production efficiency of agricultural ecosystems.

## Supplementary Information

**Additional file 1.**

## Data Availability

All data generated or analysed during this study are included in this published article and its supplementary information files. The datasets used and/or analysed during the current study are available from the corresponding author on reasonable request.

## Abbreviations

LC-MS: Liquid chromatograph-mass spectrometer
Pn: Net photosynthetic rate
PI_ABS_: Performance index based on the absorption of light
N: Nitrogen
GC-MS: Gas chromatograph-mass spectrometer
Gs: Stomatal conductance
Tr: Transpiration rate
ABS: Light energy absorption yield
ET: Electron transfer rate
DI: Heat dissipation
TR/ABS: Maximum photochemical efficiency
ABS/RC: Light energy absorbed per unit reaction center.
DI/RC: The energy dissipated per unit reaction center
ABS/CSo: Light energy absorbed per unit area
DI/CSo: Heat dissipation per unit area
RC/CSo: PSII reaction center opening ratio
ETo/RC: Electron transport efficiency per unit reaction center
ETo/CSo: Electron transport efficiency per unit area
GIP: Genetic information processing
EIP: Environmental information processing
PI_ABS_: Performance index based on absorbed light energy
