# Supplementary material for: The effects of tea plants-soybean intercropping on the secondary metabolites of tea plants by metabolomics analysis
Source: BMC Plant Biol. 2021 Oct 22;21:482. doi: 10.1186/s12870-021-03258-1 (PMC8532361; doi:10.1186/s12870-021-03258-1)
Supplement: Supplementary file 1 — Additional file 1. [file 12870_2021_3258_MOESM1_ESM.docx]

**Supplemental files:**

| **Table s1 the metabolites and their class of the monoculture and intercropping tea plants in soybean different growth stages** | | |
| --- | --- | --- |
| **The treatment of soybean different growth stage** | **Compounds** | **Class** |
| Ms-vs-Is | 2,6-Diaminooimelic acid | Amino acid derivatives |
|  | 4-Acetamidobutyric acid | Organic acids |
|  | N-Feruloyl agmatine | Phenolamides |
|  | Epicatechin gallate (ECG) | Catechin derivatives |
|  | Kaempferol 3-O-glucoside (Astragalin) | Flavonol |
|  | L-Saccharopine | Amino acid derivatives |
|  | Neochlorogenic acid (5-O-Caffeoylquinic acid) | Quinate and its derivatives |
|  | Kaempferol 3-O-galactoside (Trifolin) | Flavonol |
|  | Salicylic acid (SA) | Phytohormones |
| Mf-vs-If | 5-O-p-Coumaroylquinic acid | Quinate and its derivatives |
|  | Theobromine | Alkaloids |
|  | L-Epicatechin | Catechin derivatives |
|  | Myricetin 3-O-galactoside | Flavonol |
|  | Hesperetin 7-rutinoside (Hesperidin) | Flavanone |
|  | Hesperetin 7-O-neohesperidoside (Neohesperidin) | Flavanone |
|  | L-Saccharopine | Amino acid derivatives |
|  | Kynurenic acid | Organic acids |
|  | Luteolin C-hexoside | Flavone C-glycosides |
|  | Dihydromyricetin | Flavonol |
|  | Isohemiphloin | Alkaloids |
|  | Quercetin 3-O-glucoside (Isotrifoliin) | Flavonol |
|  | Chrysoeriol O-glucuronic acid | Flavone |
|  | Myricetin | Flavonol |
|  | Tricin | Flavone |
|  | Gallic acid | Benzoic acid derivatives |
| Mm-vs-Im | 2,6-Diaminooimelic acid | Amino acid derivatives |
|  | Acetylcholine | Cholines |
|  | D-Sorbitol | Alcohols and polyols |
|  | Pyridoxine | Vitamins |
|  | N-Feruloyl putrescine | Phenolamides |
|  | Salicylic acid (SA) | Phytohormones |
|  | Nα-Acetyl-L-glutamine | Amino acid derivatives |
|  | LysoPC 18:3 | Lipids-Glycerophospholipids |
|  | Epigallate catechin gallate (EGCG) | Catechin derivatives |
|  | Quinic acid | Quinate and its derivatives |
|  | 2-Methylsuccinic acid | Organic acids |
|  | Kaempferol 3-O-glucoside (Astragalin) | Flavonol |
|  | LysoPC 16:0 (2n isomer) | Lipids-Glycerophospholipids |
|  | Isohemiphloin | Alkaloids |
|  | LysoPC 16:0 | Lipids-Glycerophospholipids |
|  | Citric acid | Organic acids |
|  | L-Theanine | Amino acids |

| **Table s2 the metabolites involved in membrane transport of the EIP** | | | | |
| --- | --- | --- | --- | --- |
| **ID** | **Molecular Weight (Da)** | **Ionization model** | **Compounds** | **Class** |
|  |  |  |  |  |
| Cam131 | 131.0946 | [M+H]+ | L-Isoleucine | Amino acids |
| Cam15 | 146.1055 | [M+H]+ | L-(+)-Lysine | Amino acids |
| Cam16 | 155.0695 | [M+H]+ | L-Histidine | Amino acids |
| Cam196 | 165.079 | [M+H]+ | L-Phenylalanine | Amino acids |
| Cam34 | 119.0582 | [M+H]+ | L-Threonine | Amino acids |
| Cam36 | 146.069 | [M+H]+ | L-Glutamine | Amino acids |
| Cam47 | 133.0375 | [M+H]+ | L-Aspartic acid | Amino acids |
| Cam48 | 147.0532 | [M+H]+ | L-Glutamic acid | Amino acids |
| Cam65 | 115.0633 | [M+H]+ | L-Proline | Amino acids |
| Cam78 | 117.079 | [M+H]+ | L-Valine | Amino acids |
| Cam784 | 174.1117 | [M-H]- | L-(+)-Arginine | Amino acids |
| Cam792 | 105.0426 | [M-H]- | L-Serine | Amino acids |
| Cam925 | 131.0946 | [M-H]- | L-Leucine | Amino acids |
| Cam32 | 131.1 | [M+H]+ | 5-Aminolevulinate | Organic acids |
| Cam92 | 97.9 | [M+H]+ | Phosphoric acid | Organic acids |
| Cam794 | 125.015 | [M-H]- | 2-Aminoethanesulfonic acid | Organic acids |
| Cam1066 | 166.0266 | [M-H]- | Phthalic acid | Organic acids |
| Cam2 | 145.2 | [M+H]+ | Spermidine | Phenolamides |
| Cam7 | 88.1 | [M+H]+ | Putrescine | Phenolamides |
| Cam55 | 182.079 | [M+H]+ | D-Sorbitol | Alcohols and polyols |
| Cam56 | 182.079 | [M+H]+ | D-Mannitol | Alcohols and polyols |
| Cam819 | 180.063 | [M-H]- | D(+)-Glucose | Carbohydrates |
| Cam828 | 342.11621 | [M-H]- | D-(+)-Sucrose | Carbohydrates |
| Cam86 | 221.09 | [M+H]+ | N-Acetyl-D-glucosamine | Carbohydrates |
| Cam355 | 244.088 | [M+H]+ | Biotin | Vitamins |
| Cam789 | 264.1 | [M-H]- | Thiamine | Vitamins |
| Cam59 | 103.1 | [M+H]+ | Choline | Cholines |
| Cam80 | 117.079 | [M+H]+ | Betaine | Alkaloids |
| Cam791 | 180.0634 | [M-H]- | Inositol | Others |
| Cam406 | 125.1 | [M+H]+ | 2-Aminoethylphosphonate | Others |
